# Tumoricidal Activity of RNase A and DNase I

**Published:** 2010-04

**Authors:** O.A. Patutina, N.L. Mironova, E.I. Ryabchikova, N.A. Popova, V.P. Nikolin, V.I. Kaledin, V.V. Vlassov, M.A. Zenkova

**Affiliations:** Institute of Chemical Biology and Fundamental Medicine, Siberian Branch, Russian Academy of Sciences; Institute of Cytology and Genetics, Siberian Branch, Russian Academy of Sciences

**Keywords:** antimetastatic activity, DNase I, RNase A, Lewis lung carcinoma, hepatoma 1A

## Abstract

In our work the antitumor and antimetastatic activities of RNase A and DNase I were studied using two murine models of pulmonary (Lewis lung carcinoma) and liver (hepatoma A–1) metastases. We found that intramuscular administration of RNase A at the dose range of 0.1–50 µ g/kg retarded the primary tumor growth by 20–40%, and this effect disappeared with the increase in RNase A dose over 0.5 mg/kg. DNase I showed no effect on the primary tumor growth. The intramuscular administration of RNase A (0.35–7 µ g/kg) or DNase I (0.02–2.3 mg/kg) resulted in a considerable decrease in the metastasis number into the lungs of animals with Lewis lung carcinoma and a decrease of the hepatic index of animals with hepatoma 1A. A histological analysis of the organs occupied by metastases revealed that the administration of RNase A and DNase I induced metastasis pathomorphism as manifested by the destruction of oncocytes, an increase in necrosis and apoptosis foci in metastases, and mononuclear infiltration. Our data indicated that RNase A and DNase I are highly promising as supplementary therapeutics for the treatment of metastasizing tumors.

## INTRODUCTION

Recent data on the implication of small noncoding RNAs in tumorigenesis [1–3] and tumor–derived DNAs in metastasis progression (genometastasis hypothesis) [4] gave a new initiative to the study of enzymes cleaving nucleic acids as potential antitumor and antimetastatic agents.

Extensive studies on the antitumor potential of exogenous ribonucleases are being conducted worldwide. The high antitumor activity of the RNase A family members BS–RNase [5–8] and onconase [9–11] has been shown. Of this family, RNase A was first studied for antitumor activity [12–14]. The data of these experiments were contradictory. Some authors reported high antitumor activity in RNase A [12, 13], whereas others reported its complete absence [14, 15]. The absence of any antitumor effect of RNase A was attributed to its inactivation by ribonuclease inhibitor [16, 17]; both onconase and BS–RNase can avoid interaction with the inhibitor, thus keeping their cytotoxic activity against tumor cells [18–20]. The antimetastatic potential of DNase I was demonstrated * in vivo* using a L5178Y–ML liver metastasis model [21, 22]. However, the use of DNase I as an adjuvant in cancer therapy was not further extended.

In this work we studied the antitumor and antimetastatic effects of RNase A and DNase I on two murine tumor models: Lewis lung carcinoma (LLC) metastasizing to the lungs and hepatoma A–1 (HA–1) metastasizing to the liver. The intramuscular administration of RNase A at a dose ranging within 0.1–50 µg/kg resulted in the retardation of tumor growth by 20–40%. The administration of either RNase A or DNase I led to a two– to threefold decrease in the number of metastases in the lungs (LLC) or a decrease of the hepatic index (HA–1). A histological analysis revealed the destruction of tumor cells, an increase in the number of necrotic and apoptotic sections in metastatic foci, and mononuclear infiltration following treatment with the enzymes.

## MATERIALS AND METHODS

RNase A (mol. wt 13,700) and DNase I (2.155 kU/mg) from bovine pancreas **** were purchased from Sigma (United States); [ γ –^32^P]adenosine–5’–triphosphate ([γ –^32^P]ATP) (3,000 Ci/mmole) was purchased from Biosan (Russia), and T4 polynucleotide kinase was purchased from Fermentas (Lithuania). The pHIV–2 plasmid was kindly provided by Prof. Hans J. Gross (University of Wuerzburg, Wuerzburg, Germany).

LLC and HA–1 tumor strains were obtained from the vivarium at the Institute of Cytology and Genetics, Siberian Branch, Russian Academy of Sciences (SB RAS), Novosibirsk, Russia.

The HIV–1 RNA fragment prepared by *in vitro* transcription was labeled at the 5’–end using γ –^32^P ATP and T4–polynucleotide kinase [23].

**Determination of RNase A activity**. A reaction mixture (10 µl total volume) containing 50 000 cpm of 5’–[^32^P]–labeled RNA, 10^–10^–10^–7^M RNase A, 50 mM Tris–HCl, pH 7.0, 200 mM KCl, 1 mM EDTA, and 100 µg/ml of RNA carrier was incubated at 37°C for 1–15 min. Following incubation, the reaction mixtures were extracted with phenol and RNA was precipitated from an aqueous phase with 96% ethanol. The products of RNA cleavage were analyzed by electrophoresis in 12% denaturing polyacrylamide gel.

**Determination of DNase I activity**. A reaction mixture (1 0 µ l total volume) containing 0.2 µg of pHIV–2 plasmid DNA, 0.01–1 U of DNase I, 10 mM Tris–HCl, pH 7.5, 2.5 mM MgCl_2_, and 0.1 mM CaCl_2_ was incubated at 37°C for 1–15 min. The reaction was quenched by heating at 60°C for 10 min. The products of DNA cleavage were analyzed by electrophoresis in 1% agarose gel.

**Tumor models**. Female C57Bl/6 mice (10–11 week–old) and female A/Sn mice (12–14 week–old) were housed in plastic cages (8–10 animals per cage) under normal daylight conditions. Water and food were provided ad libitum. All procedures with the animals were carried out according to approved methods and recommendations for laboratory–animal care [European Communities Council Directive 86/609/CEE].

Solid LLC or HA–1 tumor development was generated by injecting corresponding tumor cells (10^6^ cells per animal) into the femoral muscle of С 57Bl/6J or A/Sn mice, respectively.

**Intramuscular administration of RNase A and DNase I and an examination of their effect on the primary tumor and metastases**. On day 4 or 8 after the implantation of LLCtumor cells, C57Bl/6J mice were divided into groups and intramuscular injections were performed daily as follows: group 1 (control) received saline and groups 2–9 received 0.1 ml of RNase A saline solution (0.1, 0.5, 1, 10, and 50 µg/kg and 0.5, 1 and 10 mg/kg, respectively); groups 10–13 received 0.1 ml of DNase I saline solution (0.02, 0.23, 1.15, and 2.3 mg/kg, respectively).

On day 8 after the implantation of НА –1 tumor cells, A/Sn mice were divided into groups and intramuscular injections were performed daily as follows: group 1 (the control) received saline and groups 2–4 received 0.1 ml of RNase A saline solution (0.35, 0.7 and 7 µg/kg, respectively); groups 5–9 received 0.1 ml of DNase I saline solution (0.02, 0.23, 1.15, and 2.3 mg/kg, respectively).

During the experiment, animals were injected 8–10 times with either enzyme solution or saline. The tumor size was measured every three days with calipers, and the tumor volume was calculated from the equation V = (π/6 × length × width × height) [24].

On day 20 after tumor implantation, the mice were killed by cervical dislocation . Livers of A/Sn mice with HA–1 were weighed, and the hepatic index (HI) was calculated from the equation HI = (liver weight/body weight) × 100%. The average liver increment (ALI) during tumor development was calculated by the subtraction of healthy animals’ HI (4.5% for A/Sn mice) from the mean HI of the experimental group. Therapeutic efficacy (TE) was calculated from the equation TE (%) = 100 – ALI_exp_ / ALI_control_ × 100%.

The lungs of animals with LLC and liver of animals with НА –1 were fixed in 4% formaldehyde for further histological analysis. The number of metastases in the lungs of LLC–bearing mice was enumerated using a binocular microscope.

**Histological analysis**. Fixed lungs and liver were treated according to routine protocol and embedded in paraffin. Histological sections (5–µm–thick) were prepared on a microtome and stained with hematoxylin and eosin (HE staining). Pathomorphological features were evaluated visually using an Axioimager Z microscope (Zeiss).

**Statistical analysis**. Whenever the data showed normal distribution, their statistical processing was performed using Student’s *t*–test. Otherwise, the Mann–Whitney nonparametric statistics was used. Differences were regarded as significant at p < 0.05.

## RESULTS AND DISCUSSION

**Choice of Dose Ranges for RNase A and DNase I Used in Experiments *In Vivo***. Since the enzymatic activities of RNase A and DNase I were assumed to be essential for the antitumor effect of these enzymes, concentrations which provide a 50% cleavage of substrates in a relatively short time were determined in experiments *in vitro*.

To do this, [5’–^32^P]RNA (10^–5^M) was incubated in the presence of 10^–10^–10^–7^M RNase A at 37°C for 1–15 min. Kinetics of RNA cleavage has shown a 50% cleavage of the substrate in 10 min at a RNase A concentration of 10^–9 ^M. Similarly, 50% cleavage of DNA substrate was achieved in 1 min by 10 U of DNase I per ml. These concentrations of RNase A and DNase I were taken as the starting points to select the appropriate doses of the enzymes for the *in vivo* assay.

## Effects of rnase a and dnase i on primary tumor growth.

**Intramuscular administration of RNase A to LLC–bearing C57Bl/6J mice**. The effect of RNase A on the primary tumor growth was examined in experiments with LLC–bearing C57Bl/6J mice. On day 4 after tumor transplantation, the animals began receiving daily intramuscular injections of a saline (control) or RNase solution ranging in concentration from 0.1 µg to 10 mg per kg of body weight (experiment).

**Fig. 1:**
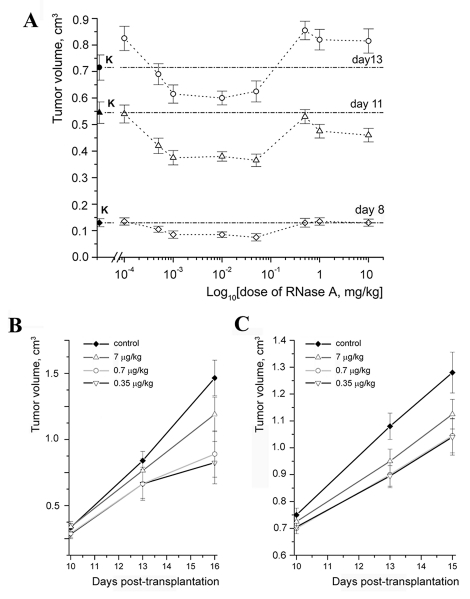
Antitumor effect of RNAse A. A. The effect of RNase A on the growth of a primary LLC tumor in C57Bl/6J mice (concentration depen- dence). B. The effect of RNase A in 0.35, 0.7 and 7 µg/kg dosages on the growth rate of a primary LLC tumor in C57Bl/6J mice. c. The effect of RNase A in 0.35, 0.7 and 7 µg /kg dosages on the growth rate of a primary HA-1 tumor in A/Sn mice.

Figure 1A demonstrates changes in the size of tumors during the experiment depending on the RNase A dose. One can see a retardation of tumor growth in the LLC–bearing animals treated with RNase A at a dose ranging within 0.5–50 µg/kg. On day 8 after LLC transplantation, the tumor volume in these experimental groups was retarded by 20–40% when compared with the control. This difference was 23–33% on day 11 and 16% on day 13. No effect on tumor growth was observed in animal groups treated with RNase A at a dose above 0.5 mg/kg (Fig. 1A).

**Intramuscular administration of RNase A to HA–1–bearing A/Sn mice**. To ensure that the antitumor activity of RNase A is not tumor–specific, we examined it on another model, hepatoma A1 in A/Sn mice. Since RNase A showed a marked activity on the LLC model at a dose ranging within 0.5–50 µg/kg, we also used this dose range in experiments with HA–1. The LLC–bearing С 57Bl/6J mice were used as positive controls in these experiments. Beginning from day 8 after tumor implantation, when the tumors became palpable, the mice with HA–1 or LLCreceived intramuscular injections of either a saline or RNase A solution at doses of 0.35, 0.7, and 7 µg/kg.

A comparison of tumor sizes in the control group and groups of animals with either LLC or HA–1 treated with RNase A showed an insignificant difference between the groups at the initial stage of treatment (day 10 after tumor transplantation) (Figs. 1B, 1C). On day 15 the tumor size in the groups of animals with HA–1 treated with RNase A at doses of 0.35 and 0.7 µg/kg was 23% less than that in the control (Fig. 1C); in the groups of animals with LLC, it was 43% less (Fig. 1B). It is worth noting that the antitumor effect of RNase A on the LLC model did not depend on which day (4 or 8) the treatment began after implantation.

**Intramuscular administration of DNase I to LLC–bearing C57Bl/6J mice and HA–1–bearing A/Sn mice. The antitumor potential of DNase I was evaluated on two tumor models, LLC and HA–1**. Starting at day 8 after the implantation of LLC to C57Bl/6J mice and HA–1 to A/Sn mice, the animals were injected with DNase I at a dose ranging within 0.02–2.3 mg/kg. Measuring the tumor size showed that the injection of DNase I does not lead to the retardation of primary tumor growth.

## Effects of rnase a and dnase i on metastasis development.

The antimetastatic activities of RNase A and DNase I (their capability to decrease the number of metastases in target organs) were estimated from (1) a histological analysis of target organs (the lungs for LLC and liver for HA–1), (2) a microscopic examination of the metastasis number in the lungs of LLC–bearing animals, and (3) the liver weight alteration (hepatic index) in animals with HA–1.

## A histological analysis of metastases in the lungs of animals with LLC and in the liver of animals with HA-1.

**Fig. 2:**
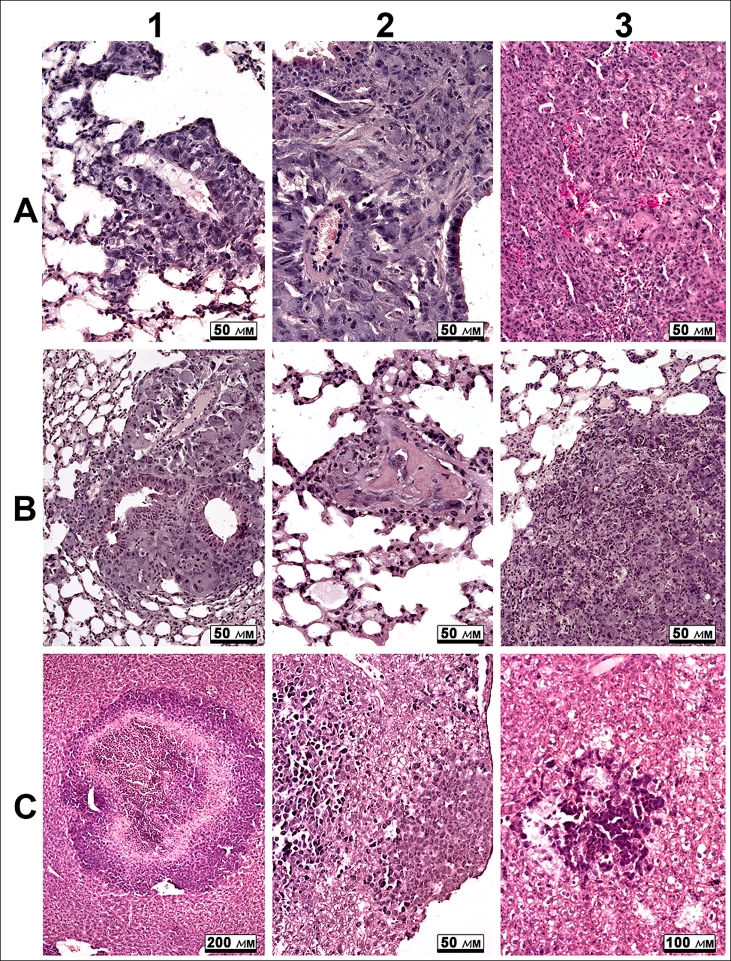
A. Metastases in the lungs of animals with LLC (A1 and A2) tumors and in the liver of animals with НА-1 (A3). B. Metastases in the lungs of animals with LLC tumors after treatment with DNase I (0.12 mg/kg) (B1) and RNase А (0.7 µg/kg) (B2 and B3). c. Metastasis in the liver of animals with HA-1 tumors after treatment with DNase I (0.02 mg/kg) (C1), DNase I (1.2 mg/kg) (C2) and RNase А (0.35 µg/kg) (C3)

Metastasis formation in the pulmonary tissue is a characteristic feature of LLC. Distinct metastases and multiple groups of tumor cells were observed in the lungs of the control mice (Fig. 2A1, 2A2). Metastases of different sizes and irregular shapes were predominantly localized in the subpleural area. Some signs of mononuclear infiltration were observed in large metastases extending over several bronchi and large vessels (Fig. 2A1). Surface metastases were composed of two or three layers of tumor cells expanding along the pleura.

The development of heavy metastases in the liver is a characteristic feature of HA–1 progression. A multitude of metastases of different sizes are found on histological sections of liver tissue (Fig. 2A3). We have revealed several morphologic types of metastases, such as (1) distinctly bordered metastases with pseudoglandular structures at the periphery of basophilic cells with pale densely packed cells at the center; (2) loose accumulations of basophilic oncocytes under hepatic capsule, and (3) small loose aggregations composed of dark basophilic oncocytes. Numerous mitoses in metastases, individual disseminated tumor cells, the lymphocyte infiltration of liver parenchyma, and dystrophic changes and necroses of hepatocytes were observed in liver tissue of mice with HA–1 (Fig. 2A3).

## A histological analysis of metastases in the lungs of animals with LLC and in the liver of animals with HA-1 treated with enzymes.

The administration of RNase A or DNase I to animals with LLC induced dystrophic changes in metastases in the lungs (Fig. 2B). The morphologic parameters of these changes were identical in all groups irrespective of the dose: an increase in the number of necroses and apoptoses, a dystrophic transformation of oncocytes, and a considerable mononuclear infiltration of tumor extravasates and metastases (Fig. 2B, 1–3).

A histological analysis of the metastases in the liver tissue of mice with HA–1 treated with RNase A or DNase I at different doses has shown clear morphologic changes with similar features. Both central and perifocal necroses, tissue edema, numerous hemorrhages, and clear mononuclear infiltration were observed in metastatic foci (Fig. 2C, 1–3). It should be noted that, unlike control animals, in which tumor infiltrates were found in the myocardium and kidney, metastases were not found in these organs of mice with HA–1 treated with the enzymes.

The state of immunity organs of animals with HA–1 also came under our notice. In particular, we observed some signs of the accidental involution of thymus, such as an increase in the amount of lymphocytes in the medulla or even an inversion of the thymus layers. Similar alterations suggesting expressed antigenic stimulation were found in the spleen. The degree of manifestation of these signs of antigenic stimulation correlated with the enzyme dose.

Thus, a comparison between control animals with LLC or HA–1 and experimental ones treated with RNase A or DNase I has shown signs of induced pathomorphism of metastases manifested as the expressed dystrophic involution of tumor cells and an intensification of mononuclear infiltration.

## Counting metastases in the lungs of mice with LLC following treatment with enzymes.

A microscopic examination of metastases on the surface of the LLC–bearing mouse lungs has shown that treating these animals with enzymes leads to a significant decrease in the metastasis number. The average number of metastases in groups of LLC–bearing mice treated with RNase A at doses of 0.5 µg/kg, 0.7 µg/kg, and 10 mg/kg were 14 ± 3, 15 ± 4, and 18 ± 4, respectively. The average number of metastases in groups of LLC–bearing mice treated with DNase I at doses of 0.02, 0.12, and 2.3 mg/kg were 10 ± 4, 16 ± 7, and 18 ± 4, respectively, whereas in the untreated animal group this amount was 30 ± 5. Thus, the observed amount of metastases in groups of LLC–bearing mice treated with the enzymes was two– to threefold less than in the control.

**Fig. 3:**
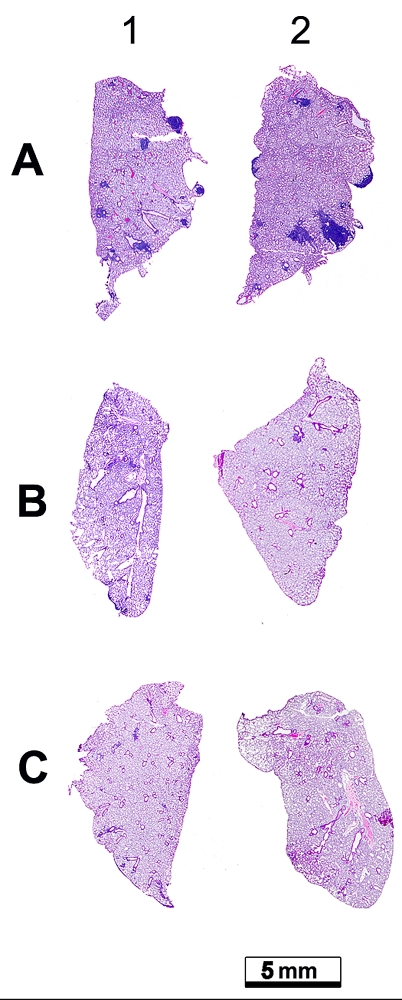
Histotopogramme of the lung lobes of C57Bl/6 mice with LLC. A. Animals which received injections of normal saline solution B. Animals which received injections of DNase I at a dosage of 0.02 mg/kg. C. Animals which received injections of RNase A at a dosage of 0.7 µg/kg. Stained by hematoxylin and eosine.

An analysis of metastases in the lungs of LLC–bearing animals has shown not only morphologic changes and a decrease in their amount following treatment with the enzymes, but also an existential reduction of the metastasis area and an altered localization in the organ. Figure 3 shows the lungs of LLC–bearing animals without treatment (Fig. 3A) and after treatment with the enzymes (Fig. 3B, 3C). The decrease in both the amounts of metastatic foci and the area of metastases is plain to see.

## Estimation of the therapeutic efficacy of enzymes in the treatment of animals with HA-1

The diffuse boundaries of metastatic foci in hepatic parenchyma made it impossible to use microscopy for counting metastases in the liver of animals bearing HA–1. Since the liver increases in weight during the metastasis development, we used the hepatic index (HI) reflecting disease severity and calculated as HI = (liver weight/body weight) × 100% to estimate the antimetastatic effects of the enzymes: the relative HI reduction in a group of treated animals compared to the control group served as the criterion of the therapeutic efficacy (TE). The data on the average liver increment (ALI) of animals with HA–1 compared to that of the healthy ones were used to estimate TE (Table 1). A noticeable decrease in HI in HA–1–bearing animals treated with the enzymes was observed relatively to the control. The TE value varied from 30% to 42% in HA–1–bearing animals treated with RNase A and from 40% to 53% in those treated with DNase I.

**Table 1:** Hepatic index (HI), average liver increment (ALI), and treatment efficiency (TE) of the A/Sn mice bearing HA-1.

	Control	Healthy mice	RNase A, μg/kg	DNase I, mg/kg
			0.35	0.7	7	0.02	0.23	0.12	2.3
^ (1) ^HI, %	6.7±0.3	4.5	5.9±0.2	6.0±0.2	5.9±0.2	5.5±0.3	5.8±0.2	5.6±0.3	5.7±0.2
^ (2) ^ALI, %	2.2	–	1.3	1.5	1.4	1.0	1.3	1.1	1.2
^ (3) ^TE, %	0	–	42	30	38	53	40	52	46

HI = (liver weight/mouse weight) × 100%;

ALI (%) = HI_experiment_ – HI_healthy_ = 4.5%;

TE (%) = 100 – ALI_treatment_ / ALI_control_ × 100.

## DISCUSSION

As was mentioned in the Introduction, the largest representative of the RNase A family, pancreatic RNase A, demonstrated weak antitumor activity at high doses (above 10 mg/kg) [14, 15] and DNase I was capable of metastasis growth suppression [21, 22].

In this work, we studied both the antitumor and antimetastatic activities of RNase A *in vivo * using doses ranging from 0.1 µg/kg to 10 mg/kg. We have shown that the intramuscular administration of RNase A at doses ranging within 0.5–50 µg/kg leads to the retardation of primary tumor growth by 20–40% with a more pronounced effect at early stages of tumor development (on the 8th day). Doses above 0.5 mg/kg, RNase A did not affect the tumor growth, which conforms to the previously reported data of other authors [17, 25]. The administration of DNase I at a dose in the range of 0.02–2.3 mg/kg did not result in any retardation of the primary tumor growth. We found that the intramuscular administration of any of these enzymes led to a considerable (two– to threefold) decrease in both the amount and size of metastases in the lungs of animals with LLC. In the case of hepatoma HA–1, the intramuscular administration of either RNase A or DNase I led to a decrease in the liver weight relatively to the control, with a therapeutic efficacy of 30–42% for RNase A and 40–53% for DNase I. A histological analysis of the lungs and liver has shown that both enzymes similarly destroy tumor cells and increase the number of necroses and apoptoses in metastatic foci. Our data make it possible for us to conclude that both enzymes have high antimetastatic activity.

Yet there is no commonly accepted mechanism of antitumor activity for ribonucleases. The antitumor effect of RNase A that we observed can occur due to (1) the degradation of encoding intracellular RNAs and, as a consequence, (a) the arrest of protein synthesis [26, 27] and (b) the alteration of gene expression profile via RNA cleavage products [28]; (2) the degradation of noncoding RNAs (pre–miRNAs, miRNAs, and siRNAs) [2, 29]; (3) the destabilization of the RNA structure [30]; (4) the blockage of RNA functions [31]; (5) the influence on signaling pathways [32–34]; and (6) the cutoff of uncontrolled potassium influx via calcium–dependent potassium channels of tumor cells [35]. Also, one cannot exclude other as of yet unknown mechanisms.

We hypothesize that the antimetastatic effects of RNase A and DNase I, as well as the antitumor effect of RNase A, are associated with the main function of these enzymes (the nucleic acid cleavage). Nevertheless, we cannot claim definitively that the antitumor effect of RNase A happens via the degradation of tumor intracellular RNAs, because a great pool of data univocally evidences for the binding of the enzyme penetrating into the cell with the ribonuclease inhibitor [17].

Putative targets for RNase A are RNAs circulating in blood plasma, including pre–miRNAs and miRNAs implicated in the control of oncogenesis and invasion [3, 36, 37]. The expression of most miRNAs implicated in the control of tumor–specific genes is known to be disordered [38, 39]. In particular, the elevation of miR–9 expression in breast cancer leads to a decrease in the E–cadherin level and invasion enhancement [40]. It was shown that the level of miR–184 possessing a stimulatory effect on the antiapoptotic and proliferative potential of tumor cells is increased in the plasma of patients with squamous cell carcinoma of thetongue [41]. Putative targets for DNase I are extracellular tumor–derived DNAs that, according to the genometastatic theory, are capable of transfection of distant cells, thus providing metastatic progression in primarily unaffected organs [4].

Some small peptides show antitumor [42] and immunostimulating [43, 44] activities at extremely low doses; however, it is not really understood how they act. We cannot exclude that the antimetastatic effects of low doses of RNase A and DNase I that we found in this study might be associated with the formation of biogenic peptides due to the proteolysis of these enzymes in blood.

The disappearance of the antitumor activity of RNase A at doses above 0.5 mg/kg or upon prolonged administration (the observed decrease of antitumor effect on day 13 of tumor development) might be associated with the specific anti–RNase A antibody production. This suggestion is supported by signs of antigenic stimulation following the administration of RNase A: there is an increase in the number of lymphocytes in the medullar layer of thymus and in the spleen, an inversion of thymus layers, and a mononuclear infiltration of metastatic foci.

## CONCLUSIONS

We have shown that the intramuscular administration of RNase A or DNase I has a systemic effect on malignant tumors, which is manifested as a retardation of tumor growth (RNase A), a decrease in the amount and area of metastases, and destructive changes in metastatic foci (both enzymes). The most effective antimetastatic doses of the enzymes had no toxic effect on animals. Our data make it possible to recommend using RNase A and DNase I in the supplementary therapy of metastasizing tumors.

## ABBREVIATIONS

LLC: Lewis lung carcinoma
HA-1: hepatoma 1A
